# Unmasking Oligosecretory multiple myeloma: a case report highlighting diagnostic pitfalls

**DOI:** 10.1093/omcr/omaf242

**Published:** 2025-11-26

**Authors:** Carlos Solórzano Flores, Adolfo Izaguirre, Evangie Bravo Monroig, Jhiamluka Solano

**Affiliations:** Faculty of Medical Sciences, National Autonomous University of Honduras (UNAH), 3RQ3+73W Calle La Salud, Edificio Administrativo, Tegucigalpa 11101, Honduras; Faculty of Medical Sciences, National Autonomous University of Honduras (UNAH), 3RQ3+73W Calle La Salud, Edificio Administrativo, Tegucigalpa 11101, Honduras; School of Medicine, Autonomous University of Guadalajara (UAG), Av Montevideo 3035, Lomas del Valle, Guadalajara, Jalisco 44100, Mexico; Department of Cardiology,York Hospital & Royal College of Physician, Wigginton Road, York YO31 8HE, United Kingdom

**Keywords:** Oligosecretory multiple myeloma, plasma cell dyscrasia, monoclonal protein, diagnostic challenge, immunofixation electrophoresis

## Abstract

Oligosecretory multiple myeloma (OSMM) is a rare subtype of plasma cell dyscrasia that poses significant diagnostic challenges due to the absence of a clear monoclonal (M) spike on serum protein electrophoresis. We report the case of a 64-year-old woman with a history of ovarian tumor who presented with progressive fatigue, weight loss, bone pain, anaemia, hypercalcemia, and renal dysfunction. Despite the absence of a definitive M-spike, further immunochemical testing revealed discrete IgG-kappa bands on immunofixation, skeletal x-rays showed extensive osteolytic lesions. A bone marrow biopsy confirmed a diagnosis of OSMM. This case highlights the importance of considering oligosecretory variants in patients with clinical and radiological features suggestive of myeloma, even when routine tests appear normal, and illustrates how comprehensive evaluation with immunofixation and bone marrow examination can prevent diagnostic delays and allow timely initiation of treatment in these diagnostically challenging cases.

## Introduction

In 2022, multiple myeloma (MM) accounted for approximately 1% of all new cancer cases but nearly 10% of all hematologic malignancies, making it the second most common blood cancer worldwide [1, 2]. MM is characterized by the clonal proliferation of plasma cells in the bone marrow, which typically secrete monoclonal immunoglobulins known as M proteins [3]. Clinically, MM often presents with the CRAB features: hypercalcemia, renal impairment, anaemia, and bone lesions, accompanied by evidence of M protein in the serum or urine [4, 5].

Oligosecretory multiple myeloma (OSMM) is a rare and diagnostically challenging subtype, representing approximately 1–5% of MM cases [6]. Patients with OSMM may lack a detectable M-spike on serum protein electrophoresis (SPEP), which can lead to delayed diagnosis or misclassification. Accurate recognition of OSMM requires a high index of suspicion and expanded diagnostic testing beyond conventional methods. This report highlights the clinical significance and diagnostic complexity of OSMM, emphasizing the importance of comprehensive evaluation in atypical presentations of plasma cell dyscrasias.

## Case report

**Figure 1:**
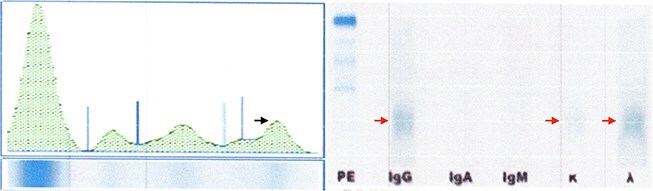
Serum protein analysis of a 64-year-old woman with oligosecretory multiple myeloma. (A) Serum protein electrophoresis showing no M spike (black arrow). (B) Immunofixation demonstrating discrete IgG and kappa light chain bands (red arrow).

A 64-year-old woman from Tegucigalpa, Honduras with type 2 diabetes underwent hysterectomy with excision of an ovarian mass, which was histopathologically confirmed as a benign serous cystadenoma. No systemic therapy was administered. She later presented with one year of generalized weakness, six months of fatigue, chest discomfort, lumbar pain, and 15 kg weight loss. Examination showed pallor and bilateral leg edema. Lab abnormalities were noted (See Table 1). Serum protein electrophoresis (SPEP) lacked a clear M spike (See Table 2), but serum immunofixation revealed discrete IgG and kappa light chain bands (See Fig. 1).

**Table 1:** Initial blood investigations.

Blood examination	Patient’s results	Normal range
White blood cells	6.1	4-10 10^3^/uL
Red blood cells	2.9	3.5-5 10^6^/uL
Haemoglobin	9.0	11.0-15.0 g/dl
Hematocrit	27.1	37.0-47.0%
Platelet count	111	150-450 10^3^/uL
Sodium	134	135-145 mmol/L
Potassium	4.2	3.5-5.0 mmol/L
Calcium	10.8	8.8-10.6 mg/dl
Glucose	187	65-110 mg/dl
Urea	92	17-43 mg/dl
Creatinine	2.45	0.50-0.90 mg/dl
Uric acid	13.06	2.3-6.1 mg/dl
Total protein	5.1	6.2-8.5 mg/dl
Albumin	2.83	3.4-4.8 g/dL
Alanine aminotransferase	23	0-31 U/L
Aspartate aminotransferase	33	0-34 U/L
Alkaline phosphatase	51	35-105 U/L
Glomerular filtration rate	21.4	>90 mL/min/1.72 m^2^

**Table 2:** Serum protein electrophoresis results.

SPEP	Patient’s results	Normal range
Total protein	4.83	6.2-8.5 mg/dl
Alpha 1	5.38	2.0-5.5%
Alpha 2	6.42	6.0-11.7%
Beta	1.45	8.2-14.5%
Gamma	9.52	9.5-19.8%
M spike	No detected	
Serum immunofixation	Discrete IgG and kappa light chain bands.	

The Bence-Jones urine test was negative. Serum immunoglobulin levels (A, G, M) were reduced (See Table 3).

**Figure 2:**
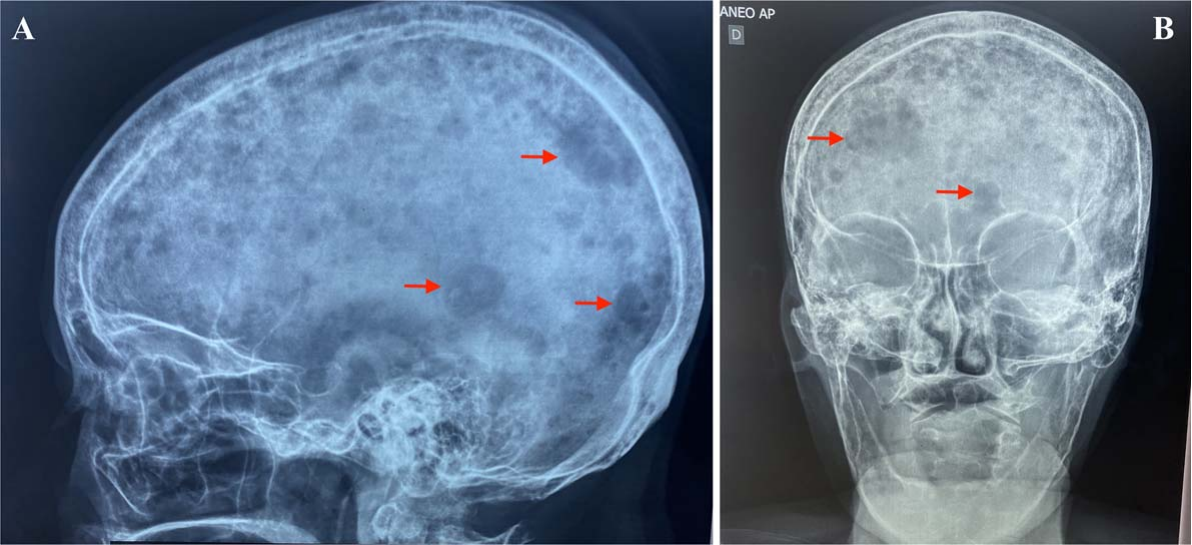
Skull radiographs of a 64-year-old woman with oligosecretory multiple myeloma. (A) Lateral view showing multiple lytic lesions. (B) Anteroposterior view highlighting lytic bone lesions (red arrows).

**Figure 3:**
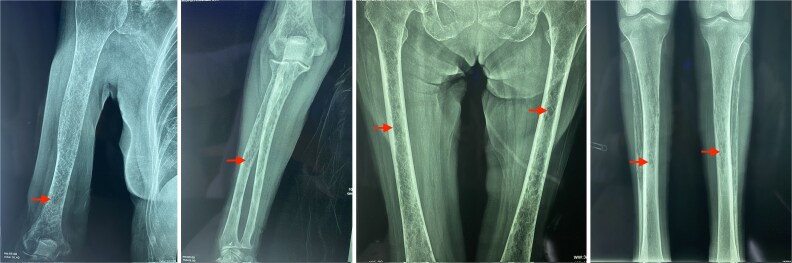
Radiographs of long bones in a 64-year-old woman with oligosecretory multiple myeloma. (A) Right humerus, (B) right ulna and radius, (C) left and right femur, (D) left and right tibia, showing multiple osteolytic lesions (red arrows).

**Figure 4:**
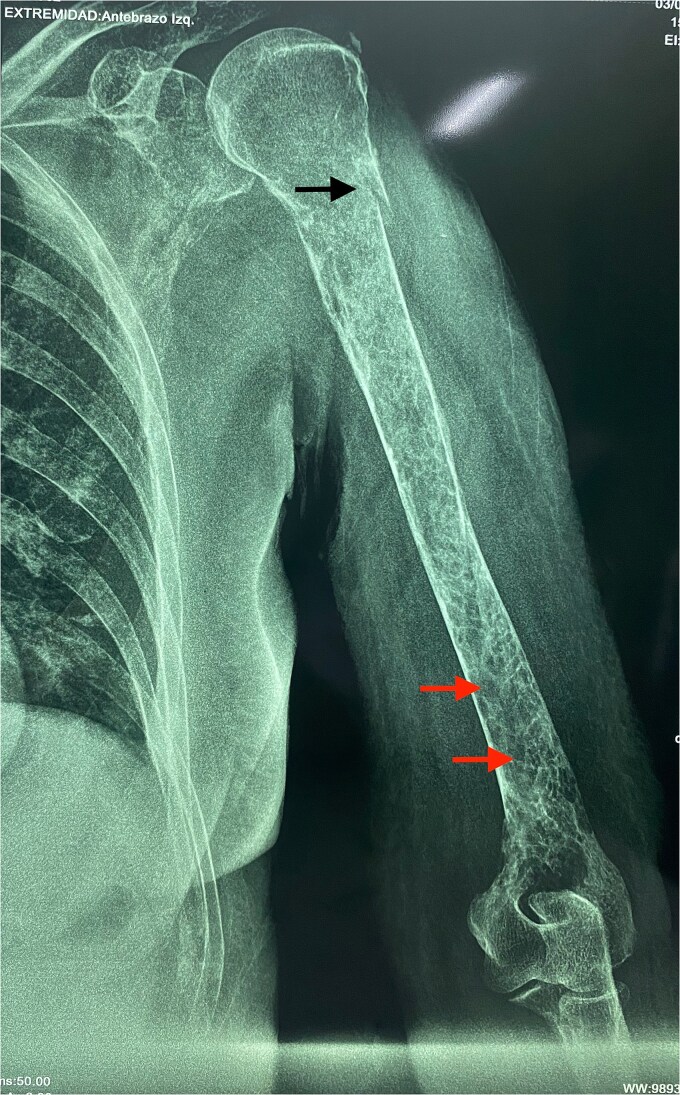
Radiograph of the left humerus in a 64-year-old woman with oligosecretory multiple myeloma, showing a fracture at the surgical neck (black arrow) and lytic lesions in the distal third of the bone (red arrows).

Although a serum free light chain (sFLC) assay could have further aided diagnosis, it was not performed due to unavailability at the public hospital and the patient’s inability to access testing elsewhere. Radiographs showed multiple lytic lesions in the skull and long bones (Figs 2 and 3), including a pathologic fracture of the left humerus (Fig. 4), spinal osteopenia, and T7–T10 compression fractures. The left arm was immobilized.

**Table 3:** Immunoglobulin a, G, M and E results.

Immunoglobulin	Patient’s results	Normal range
A	0.31	0.70-4.0 g/L
G	4.19	7.0-16.0 g/L
M	0.16	0.40-2.30 g/L
E	21.03	0.0- 100.0 UI/ml

**Figure 5:**
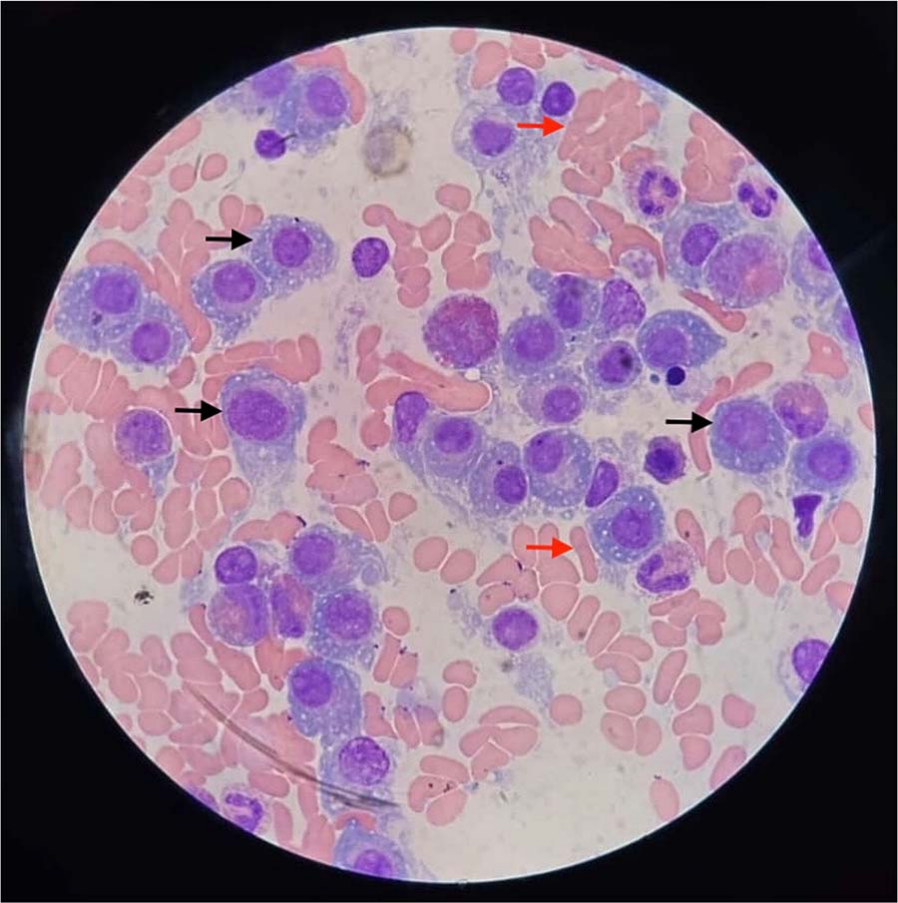
Bone marrow aspirate smear of a 64-year-old woman with oligosecretory multiple myeloma, showing numerous plasma cells (black arrows) and red blood cells in rouleaux formation (red arrows).

Bone marrow biopsy revealed 95% hypercellularity with diffuse plasma cell infiltration (Fig. 5). Flow cytometry confirmed CD138 and kappa light chain positivity, supporting a diagnosis of oligosecretory multiple myeloma (OSMM). The patient was started on a VRd regimen consisting of bortezomib 1.3 mg/m^2^ subcutaneously on days 1, 4, 8, and 11, lenalidomide 25 mg orally on days 1–14, and dexamethasone 20 mg orally on the days of and following bortezomib administration in a 21-day cycle. Cyclophosphamide 300 mg/m^2^ intravenously on days 1, 8, and 15 was added from the second cycle due to the extent of bone disease. She completed two cycles with good tolerance, experiencing only mild fatigue and peripheral neuropathy. Laboratory studies showed early subtle improvement in hemoglobin, calcium, and renal function, and her bone pain decreased. She remains under close monitoring for further treatment and disease response.

## Discussion

In this patient, the absence of a detectable M-protein initially delayed diagnosis. Plasma cell neoplasia was ultimately confirmed through discrete IgG-kappa bands on immunofixation, together with extensive lytic lesions. This case illustrates the challenges of diagnosing oligosecretory multiple myeloma (OSMM), particularly when conventional electrophoretic methods fail to detect low level M proteins.

**Figure 6:**
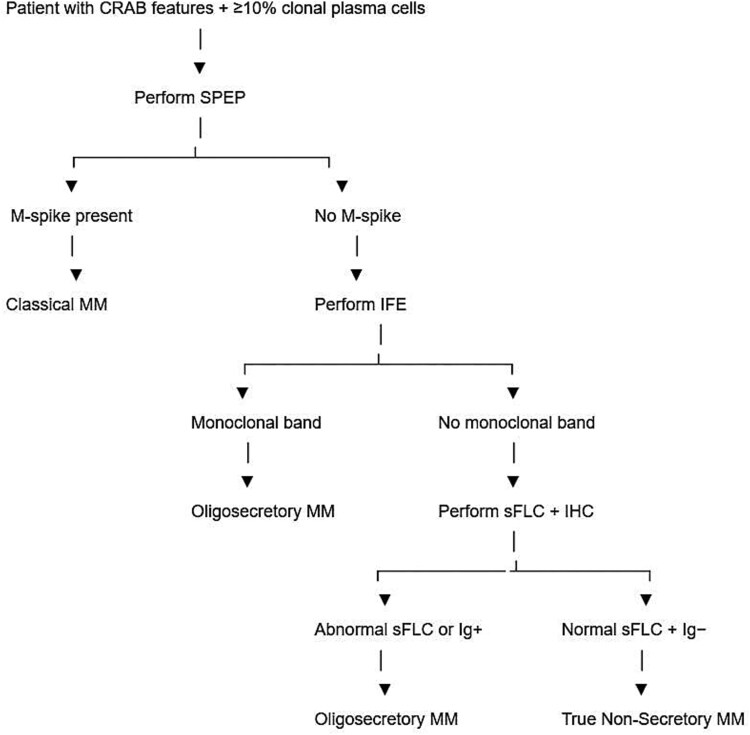
Diagnostic flow chart for MM, adapted from Charliński G, Jurczyszyn a. *non-secretory multiple myeloma: Diagnosis and management. Adv Clin Exp med.* 2021. doi: http://dx.doi.org/10.17219/acem/141455, distributed under the terms of the creative commons attribution 3.0 Unported license.

When multiple myeloma is suspected, testing should include serum protein electrophoresis (SPEP), serum immunofixation electrophoresis (SIFE), and serum free light chain (sFLC) assays [3] (Fig. 6). Approximately 3–5% of MM cases lack detectable M-protein in serum or urine [7]; most of these represent oligosecretory rather than true non-secretory MM (NSMM). Unlike classical secretory MM, where circulating paraproteins mediate end-organ damage such as renal impairment, amyloidosis, or hyperviscosity [8], NSMM and OSMM, more commonly present with bone-related symptoms and marrow infiltration [9, 10]. Pathogenetically, secretory MM involves intact immunoglobulin synthesis and secretion, whereas NSMM and OSMM may produce immunoglobulins with defective secretion or misfolded light chains [9].

Classical MM requires detectable M-protein (≥1 g/dL serum or ≥ 200 mg/day urine) [3]. OSMM lies in a diagnostic grey zone between secretory MM and true NSMM. SIFE and sFLC are essential in SPEP-negative cases. The biology behind M-protein suppression is unclear; true NSMM may involve complete loss of Ig secretion.

Our patient met diagnostic criteria: extensive osteolytic lesions, elevated creatinine (2.45 mg/dL), and anaemia (Hb 9 g/dL). Age also supported diagnosis, as over 90% of MM occurs in those > 50 years [7]. Despite this, diagnosis was delayed due to lack of M-spike, common in NSMM variants. This highlights the need for high clinical suspicion in older patients with unexplained systemic symptoms—even without paraproteinemia.

Several confounders further complicated the diagnosis. A recent ovarian tumor may have biased initial diagnostic thinking, while nonspecific symptoms such as fatigue and weight loss are common across chronic illnesses. Bone lesions prompted differential diagnoses including metastases, benign lesions, and osteoporotic fractures. Only after targeted immunochemical testing (Fig. 6) was OSMM confirmed through IgG-kappa bands, MDEs, and bone marrow evaluation.

This case underscores key learning points: OSMM can present with classic clinical and radiologic features despite negative SPEP, delaying diagnosis; serum immunofixation and bone marrow biopsy are essential for confirmation; and clinicians should maintain high suspicion in older patients with unexplained systemic or skeletal symptoms, especially where sFLC assays are unavailable. Reporting such cases emphasizes the importance of comprehensive evaluation to ensure timely diagnosis and treatment.
